# Can Polymer Helicity Affect Topological Chirality of Polymer Knots?

**DOI:** 10.1021/acsmacrolett.2c00600

**Published:** 2023-01-27

**Authors:** Yani Zhao, Jan Rothörl, Pol Besenius, Peter Virnau, Kostas Ch. Daoulas

**Affiliations:** † Max Planck Institute for Polymer Research, Ackermannweg 10, 55128 Mainz, Germany; ‡ Department of Physics, Johannes Gutenberg University Mainz, Staudingerweg 9, 55128 Mainz, Germany; § Department of Chemistry, Johannes Gutenberg University Mainz, Duesbergweg 10-14, 55128 Mainz, Germany

## Abstract

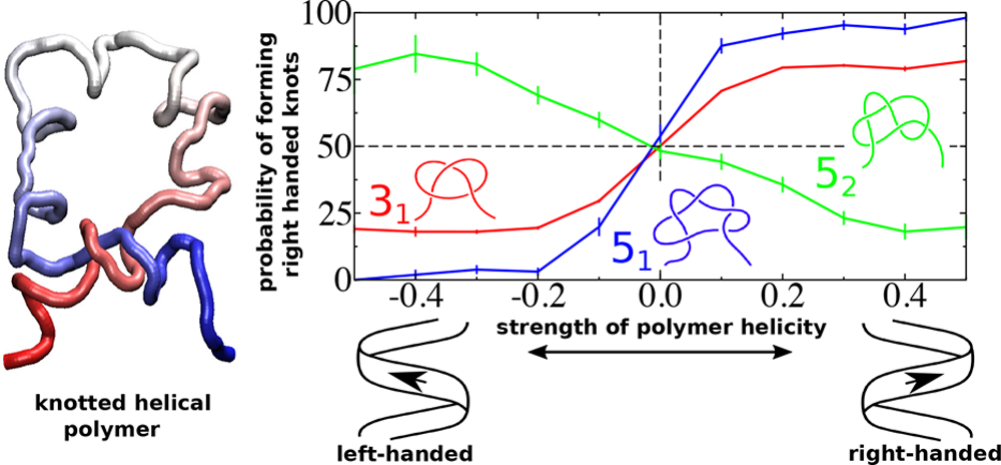

We investigate the effect of helicity in isolated polymers on the topological chirality of their knots with computer simulations. Polymers are described by generic worm-like chains (WLC), where helical conformations are promoted by chiral coupling between segments that are neighbors along the chain contour. The sign and magnitude of the coupling coefficient *u* determine the sense and strength of helicity. The corrugation of the helix is adjusted via the radius *R* of a spherical, hard excluded volume around each WLC segment. Open and compact helices are, respectively, obtained for *R* that is either zero or smaller than the length of the WLC bond, and *R* that is a few times larger than the bond length. We use a Monte Carlo algorithm to sample polymer conformations for different values of *u*, spanning the range from achiral polymers to chains with well-developed helices. Monitoring the average helix torsion and fluctuations of chiral order as a function of *u*, for two very different chain lengths, demonstrates that the coil–helix transition in this model is not a phase transition but a crossover. Statistical analysis of conformations forming the simplest chiral knots, 3_1_, 5_1_, and 5_2_, demonstrates that topological mirror symmetry is broken—knots formed by helices with a given sense prefer one handedness over the other. For the 3_1_ and 5_1_ knots, positive helical sense favors positive handedness. Intriguingly, an opposite trend is observed for 5_2_ knots, where positive helical sense promotes negative handedness. We argue that this special coupling between helicity and topological chirality stems from a generic mechanism: conformations where some of the knot crossings are found in “braids” formed by two tightly interwoven sections of the polymer.

Molecular chirality can be classified into^1−3^ geometrical, chemical, and topological chirality. Geometrical chirality applies to molecular objects that cannot^1−3^ be superimposed with their mirror image by translation and/or rotation operations. Chemical chirality incorporates the influence of actual dynamics: a molecular configuration is chemically chiral when it cannot^3^ be deformed into its mirror image by intramolecular transformations that are physically feasible under the given conditions, e.g., temperature. Topological chirality applies to molecular configurations that cannot^1−3^ be transformed into their mirror image by a continuous deformation. Here, actual dynamics is irrelevant: bond lengths, valence angles, and dihedral angles can change arbitrarily, as long as the deformed molecule does not intersect itself.

Intriguingly, polymer chains form structures that are most natural to study topological chirality: knots. Figure 1a presents the simplest possible knot, the 3_1_ (known as trefoil) knot, created, for illustration, on a generic bead–spring chain. Formally, knots are defined only in closed loops, but the concept is applicable to linear polymers after introducing an imaginary closure, indicated in Figure 1a by dashed lines. The closed 3_1_ knot is topologically chiral because it cannot^1,4,5^ be continuously transformed into its mirror image, Figure 1b. Generic cartoons in Figure 1c present the next two simplest 5_1_ and 5_2_ (known as fivefold) knots, that are chiral (we omit the achiral 4_1_ knot). Since the closure is imaginary, knots evolve, disappear, and re-emerge, as linear polymers sample their conformational space.^6^ For a polymer with chemically achiral conformations (such as the bead–spring polymer in Figure 1), one expects that knots and their mirror images are equally represented in the accessible conformational space. In other words, the mixture of knotted conformations is racemic. Conversely, the presence of chemical chirality might favor left- or right-handed knots, breaking the mirror symmetry of the knot mixture.

**Figure 1:**
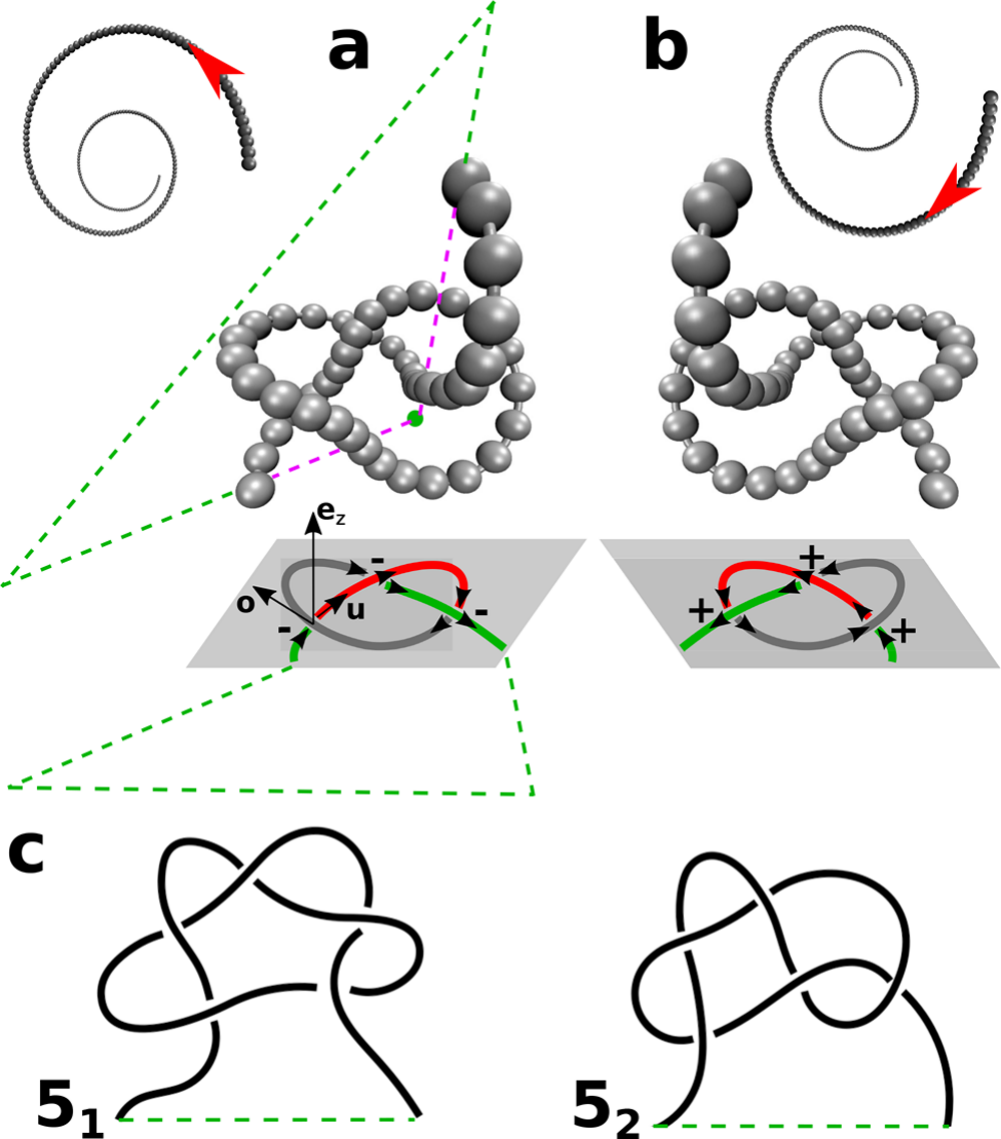
Panels (a) and (b) show, respectively, a conformation of an achiral bead–spring chain forming a left-handed 3_1_ knot and its right-handed mirror image. Panel (a) explains the closure: two lines (green dashed lines) are determined by extrapolating to infinity two line segments (dashed purple lines), connecting the center-of-mass (green circle) of the conformation with the two ends of the chain. The closure is accomplished by connecting the two extrapolated lines far from the chain (green dashed line). The two-dimensional projection of the knots on the *xy*-plane (gray) is sketched below. Red, green, and gray mark the three arcs of each projected knot. The ± signs indicate the handedness of each crossing. Panel (a) illustrates the unit vectors along *z*-axis **e**_*z*_, overpassing bond **o**, and underpassing bond **u**, used to determine the handedness of one representative crossing. The insets of panels (a) and (b) explain, respectively, the definition of a left- and right-handed helix. They show a top view of a helix which rotates into the page along the direction of the red arrow. Panel (c) shows generic sketches of the next two simplest chiral knots, 5_1_ and 5_2_ (both examples show left-handed knots).

Although this hypothesis sounds reasonable, the actual knowledge on connections between chemical and topological chirality in polymers is very limited. Among others, the influence of chirality on interactions between knots^7^ and their capability to meander through helical channels^8^ have been studied, and recently, a 8_19_ knot with controlled handedness has been created artificially.^9^ Many studies have investigated knotted structures in biopolymers such as proteins^10−15^ and DNA.^16−22^ While confining viral DNA in a capsid increases the knotting probability (≈95% for the 10 kilobase pairs (kbp) DNA strand of the P4 phage vir1 del22 tailless mutants^16,17^), knotting can be substantial even without confinement, provided that chains are long: a 166 kbp phage T4 GT7 DNA contains a knot in 70% of all cases.^21^ Although biopolymers are a canonical example of chemically chiral macromolecules, research effort concentrated on detection and classification of knots, without correlating their topology with molecular-level features.^18^ In particular, protein knots with both positive and negative handedness have been observed.^14,15^ Intriguingly, chiral DNA knots have been constructed as early as 1995 by an enzymatic closure reaction using a left-handed Z-DNA to craft trefoils with positive handedness and right-handed B-DNA for trefoils with negative handedness.^23^ However, because the knotted state was made permanent by the cyclic (closed) molecular architecture, the question regarding which knot handedness is thermodynamically favored by a right- or left-handed polymer chain was out of the scope of that study.

In this work, we consider a special, but very basic, example of chemical chirality: polymers that form right- or left-handed helices (Figure 1a and b explain the definition of a left- and right-handed helix). Our goal is to find generic relationships between helical sense and topological chirality of polymer knots.

We use a generic coarse-grained model to describe isolated helical polymer chains found in Θ solvent (ideal chains) and good solvent conditions. Polymers are represented by worm-like chains (WLC) with *N* segments (bonds). The interactions are defined through a Hamiltonian expressed in units of thermal energy *k*_B_*T* as
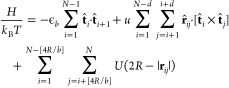
1We arbitrarily choose one of the two directions along the contour of the WLC to number the segments. Accordingly,  is a unit vector oriented along the *i*-th segment of the WLC and , where **r**_*ij*_ is the distance vector between the center-of-mass (COM) of the *i*-th and the *j*-th segment (**r**_*ij*_ is oriented from the *i*-th toward the *j*-th COM).

The first term in eq 1 manipulates polymer stiffness by adjusting the parameter ϵ_*b*_. The length of the segments is fixed to *b*. The second term is a chiral potential that couples only those segments that are separated, along the WLC contour, by less than *d* + 1 segments and favors conformations with helical twist of prescribed sense. The magnitude of *u* controls the strength of the chiral coupling and its sign determines the helical sense. Namely, *u* > 0 and *u* < 0 result, respectively, in right-handed and left-handed helices, whereas *u* = 0 corresponds to an achiral polymer. Similar chiral interactions are used in studies of cholesteric phase,^24,25^ chiral block copolymers,^26,27^ simulations of chiral liquid crystals,^28^ chiral aggregates,^29^ coil–helix transitions,^30^ and Go-like models of proteins.^31^ The third term assigns to each segment a hard excluded volume with radius *R*, centered at its COM. Specifically, *U*(2*R* – |**r**_*ij*_|) = +*∞* when 2*R* – |**r**_*ij*_| ≥ 0 and *U*(2*R* – |**r**_*ij*_|) = 0 otherwise. There are no excluded volume interactions between segments that are separated by less than [4*R*/*b*] segments along the WLC contour (angular brackets define the integer-part function). We sample the conformational space using a Monte Carlo (MC) reptation algorithm.^32^ Details are provided in the Supporting Information.

Exploring the behavior of the generic model across the four-dimensional space of parameters ϵ_*b*_, *u*, *d*, and *R* is outside the scope of this study. We choose three subsets of parameter space that are interesting for studying the behavior of knots. They have the same ϵ_*b*_ = 4 and *d* = 10 but differ regarding the size of the excluded volume: R = 0, 0.5, and 2.5, respectively (*R* is given in units of *b*). *u* is a free parameter.

For *R* = 0 and 0.5 the chosen parameters lead to open helices where the pitch *p* is substantially larger^33^ than the excluded volume of the segments, as illustrated in the main panel of Figure 2. This snapshot stems from MC simulations with *u* = 0.5. Studying knots in open helical polymers for both *R* = 0 and 0.5 is important because *R* = 0.5 retains some excluded volume. This situation might be more straightforward to realize in experiments (we provide further discussion later on). For *R* = 2.5, we obtain compact helices. The inset of Figure 2 presents a small part of a compact helix generated during MC simulations at *u* = 0.5.

**Figure 2:**
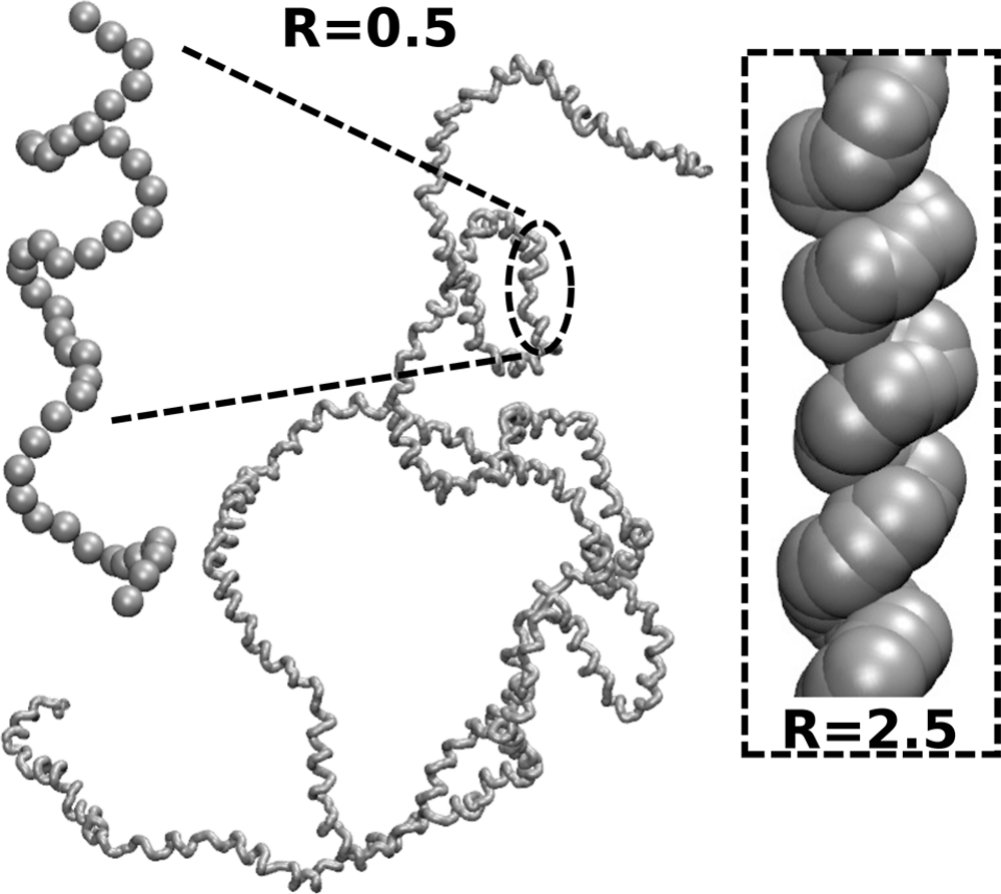
Main panel: Snapshot of an open WLC helix with *N* = 2000, ϵ_*b*_ = 4, *d* = 10, and *u* = 0.5 obtained for small radius of excluded volume *R* = 0.5. The inset presents a section of a compact WLC helix obtained for the same *N*, ϵ_*b*_, *d*, and *u* but with a large radius of excluded volume *R* = 2.5.

Before exploring the behavior of knots, we must understand how helicity changes as a function of *u*. We quantify the helicity of chains using the ensemble-averaged torsion ⟨τ⟩ of chains as an order parameter, defined as^34^
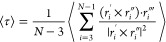
2Here *r*_*i*_ is the position vector of the *i*-th “monomer”; a WLC with *N* segments has *N* + 1 “monomers”, i.e., *N* – 1 internal junctions and two ends. The derivatives are discretized^34^ as *r*_*i*_^′^ = (*r*_*i* + 1_ − r_*i* − 1_), *r*_*i*_^″^ = *r*_*i* + 1_ – 2*r*_*i*_ + *r*_*i* – 1_, *r*_*i*_^‴^ =  (*r*_*i* + 2_ − 2(*r*_*i* + 1_ − *r*_*i* − 1_) − *r*_*i* − 2_). Figure 3a presents ⟨τ⟩ as a function of *u*, for open, *R* = 0 and 0.5, and compact, *R* = 2.5, helices. For each *R* we present plots for two representative chain lengths, *N* = 1000 and 2000. Error bars are estimated from the standard error of the mean and are smaller than symbol size. Interestingly, ⟨τ⟩ grows smoothly as *u* becomes larger, irrespective of *R*. Moreover, the plots of ⟨τ⟩ for the two different chain lengths are, practically, on top of each other; that is, we do not observe finite system-size effects. These observations suggest that in our model the transition from achiral to chiral state is not a phase transition but a continuous crossover.^35^ Formally, mirror symmetry is broken even for very small *u*, although in this limit chirality is weak.

**Figure 3:**
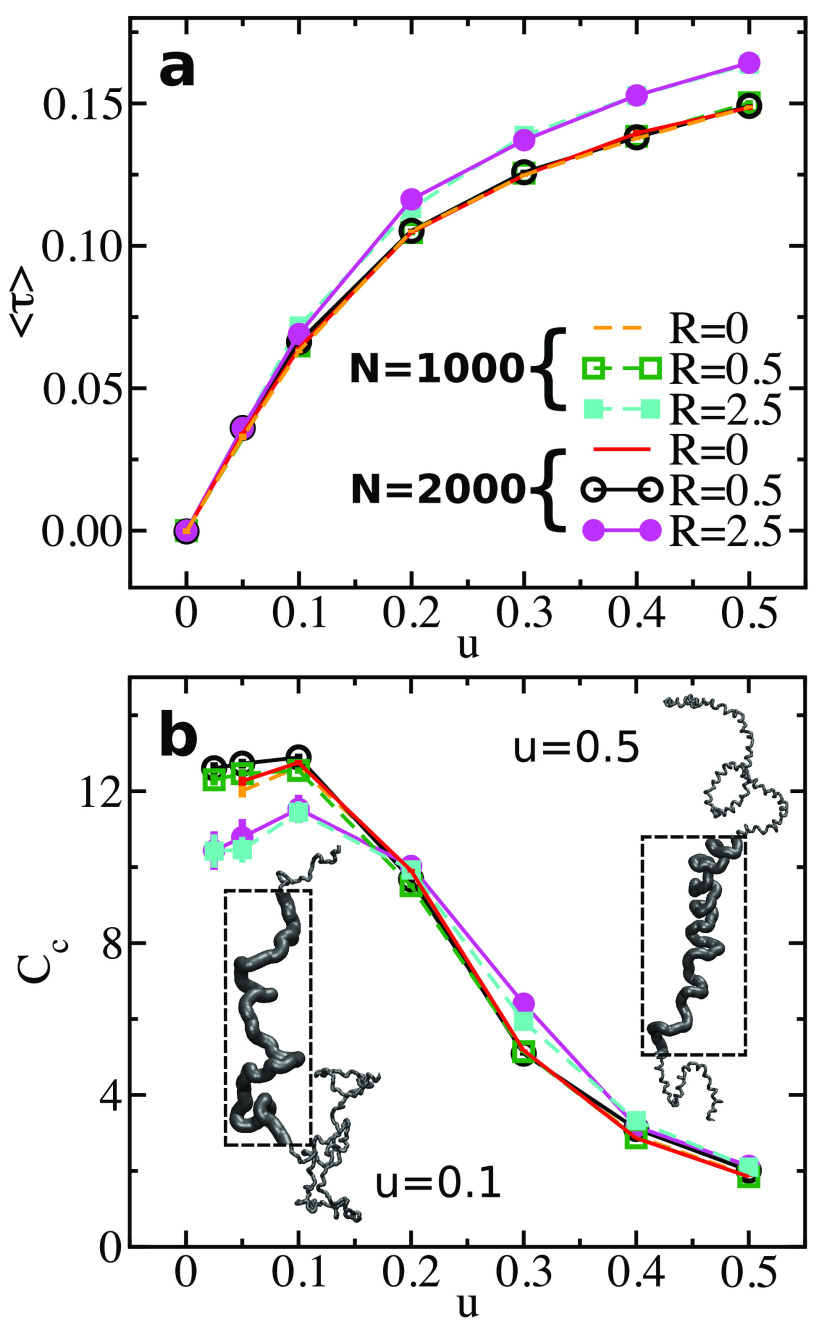
(a) Average torsion ⟨τ⟩ shown as a function of *u* for chain lengths *N* = 1000 and 2000 (dashed and solid lines). For each *N*, data corresponding to *R* = 0, 0.5, and 2.5 are presented, as indicated by the legends. (b) Variance of chiral energy *C*_c_ (normalized by *u*) presented for polymers from panel (a) as a function of *u*. The color code is the same as in panel (a). The insets show snapshots of a WLC with *N* = 1000, at *u* = 0.1 (left) and 0.5 (right). To visualize the helical structure more clearly, parts of the polymer are shown enlarged in the dashed frames.

One expects^36^ that phase transition is absent when the breaking of mirror symmetry in isolated chains is caused by chiral intramolecular interactions between a limited number of consecutive monomers. In eq 1 the chiral potential couples *d* consecutive segments only. Such potentials resemble spin-couplings in Ising-like models of helical polymers.^37−42^ They create equivalence^36,43^ to an effective one-dimensional (1D) system with local interactions, which cannot exhibit a phase transition.^44^ Some studies^35,45^ have emphasized that explaining the phenomenology of mirror-symmetry breaking in single polymers through the thermodynamics of 1D systems might be an oversimplification because, typically, there are long-range interactions between repeat units. In principle, the excluded volume interaction in eq 1 correlates segments that are far apart along the WLC contour.^46^ However, the trends in Figure 3a suggest that these correlations are not sufficient to cause a phase transition in our case.

We quantify the strength of fluctuations in chiral order through the variance of chiral energy, a heat capacity-like property, normalized by *u*:
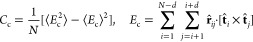
3Figure 3b presents *C*_c_ calculated for the same systems as in Figure 3a. Error bars are again small; we estimate them from the expression of the standard error of the mean for variances (“fluctuation averages”).^47^Figure 3b demonstrates that the fluctuations in chirality are stronger for small *u*. *C*_c_ exhibits a peak near *u* = 0.1, which is, however, weak. Importantly, the magnitude of the peak and the overall shape of *C*_c_ do not depend on *N* (the plots for *N* = 1000 and 2000 are practically on top of each other). This phenomenology of *C*_c_ is consistent with an absence of a phase transition. Visual inspection reveals that the stronger fluctuations at low *u* are associated with more “fluffy” conformations, exhibiting weakly helical and molten nonhelical regions. In contrast, helices are well formed for large *u* and dominate chain conformations. Figure 3b presents two snapshots of a chain with *N* = 1000 and *R* = 0.5, taken at *u* = 0.1 and 0.5.

We can now analyze knots. Generally, one can define the handedness of a knot by considering the minimal projection of the knot onto a plane.^48^ Then, the handedness of a single crossing is defined as *h* = **e**_*z*_ · ([**o** × **u**])/|[**o** × **u**]|, where **e**_*z*_, **o**, and **u** are, respectively, the unit vector along the *z*-axis, overpassing bond (at the projected crossing), and underpassing bond. The sign of the sum of all *h* in a minimal projection determines the handedness of the knot.^48,49^ For example, the minimal projection of a trefoil has only three essential crossings (see Figure 1). The sum of *h* is positive for right-handed and negative for left-handed knots.

In practice, we calculate HOMFLYPT polynomials.^50,51^ First, chain conformations, generated by MC, are “closed” by using the closure^49^ explained in Figure 1. This closure enables calculations of HOMFLYPT polynomials with the Topoly package^52^ to determine knot type and handedness.

Figure 4a presents the probability *P*_k_ (black lines) to find a knotted conformation in a *N* = 2000 chain, as a function of *u* for *R* = 0 (main panel), 0.5 (left inset), and 2.5 (right inset). The plots demonstrate that *P*_k_ is very small for chains with an excluded volume and highlight the difficulties in collecting data for analyzing knot handedness in this case. For *R* = 0 and 0.5, the *P*_k_ has a clear maximum at *u* = 0, whereas for *R* = 2.5, the plot (despite the large error bars) suggests a nonmonotonous dependence, i.e., *P*_k_ has a maximum at *u* ≠ 0. Increasing *u* makes the chains stiffer, so both trends in Figure 4a are consistent with dependencies of *P*_k_ on chain stiffness that have been reported for achiral ideal^53^ (monotonous decay) and achiral self-avoiding^54,55^ (nonmonotonous decay) chains. In Figure 4a, for *R* = 0, we separately show the probability of observing a 3_1_ (red line) knot, which is by far the most common knot type at this length scale. For *R* = 0.5 and 2.5, the preponderance of 3_1_ knots is even stronger.

**Figure 4:**
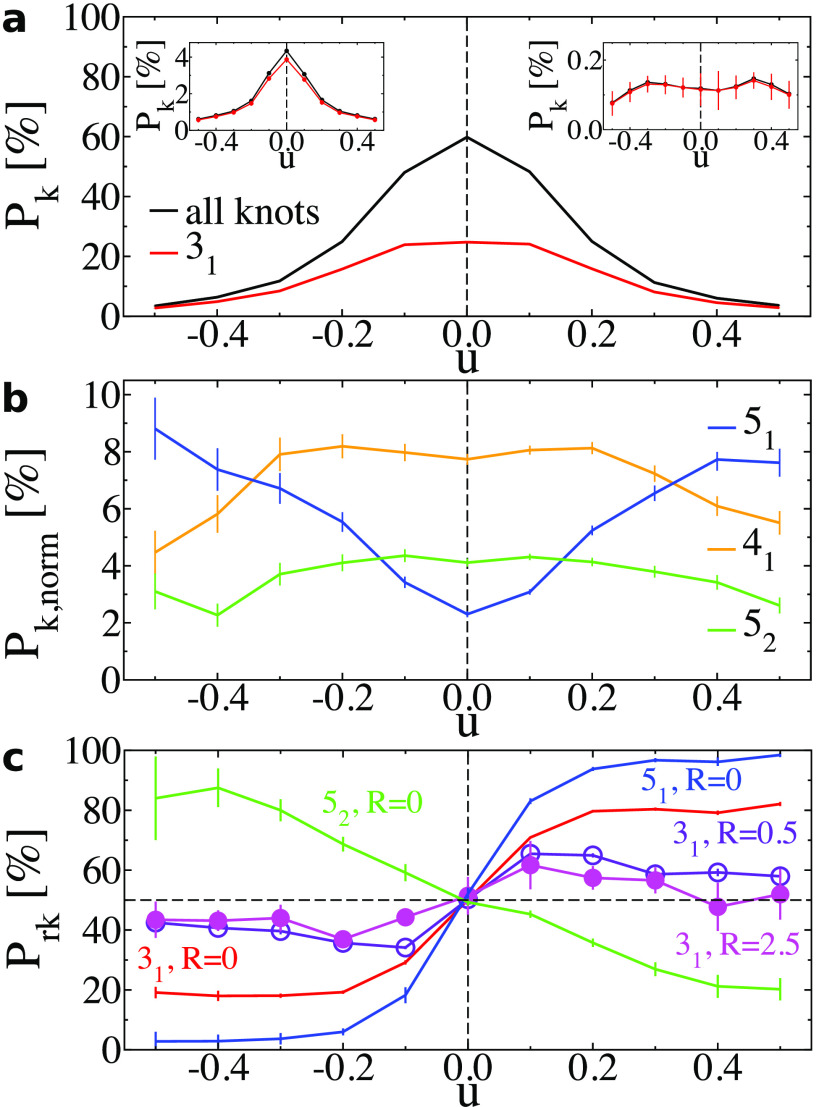
(a) The main panel shows the total knotting and occurrence probability, both indicated by *P*_k_, of 3_1_ knots as a function of *u*, for chains with *R* = 0 and *N* = 2000. Error bars are smaller than the width of the line. The left and right insets show the total knotting and occurrence probability for 3_1_ knots for *R* = 0.5 and 2.5, respectively. (b) Occurrence probability *P*_k,norm_ of 4_1_, 5_1_, and 5_2_ knots for chains with *R* = 0 and *N* = 2000 normalized by the total knotting probability. (c) Probability *P*_rk_ that a knot formed at given *u* is right handed. *P*_rk_ = 50% (horizontal black dashed line) indicates no preferred handedness. Data for 3_1_ knots obtained with *R* = 0, 0.5, and 2.5, and for fivefold knots obtained with *R* = 0 are presented, as indicated near each plot.

Figure 4b displays the probability *P*_k,norm_ of observing 4_1_ (orange line), 5_1_ (blue line), and 5_2_ (green line) knots, normalized by the total knotting probability. We use this normalization to emphasize the fraction of individual knots. The share of torus knots 3_1_ (not shown here) and 5_1_ increases with increasing |*u*|, while the portion of nontorus knots such as 4_1_ or 5_2_ is approximately constant for small |*u*| and decreases for larger |*u*|. This trend resembles results obtained for DNA confined to bacteriophage capsids^16,17^ and simulations describing such systems.^18,19^

Figure 4c is central for our work. It presents the probability *P*_rk_ that 3_1_, 5_1_, and 5_2_ knots, when formed at given *u*, are right-handed. For 3_1_ knots (red line), the mirror symmetry is clearly broken for all three *R* considered in our study, because there is an excess and depletion of right-handed knots for *u* > 0 and *u* < 0, respectively. However, there is a qualitative difference regarding how *P*_rk_ changes as a function of *u* for chains without and with excluded volume. Overall, we observe that for R = 0 the effect of helicity on handedness of knots is much stronger than for *R* > 0, that is, deviations from *P*_rk_ = 50% are more pronounced. Furthermore, *P*_rk_ increases monotonously for *R* = 0 to saturate (at least for the considered range of *u*) to a constant value. In contrast, for chains with excluded volume, the effect of helicity on knots is stronger for small *u*. Specifically, *P*_rk_ exhibits a peak near *u* = 0.1 and decays after that. Importantly, for the largest excluded volume *R* = 2.5, the excess (depletion) of right-handed knots is smaller than for *R* = 0.5.

*P*_rk_ shows broken mirror symmetry also for 5_1_ and 5_2_ knots. Because it is challenging to accumulate reliable statistics on 5_1_ and 5_2_ knots for chains with an excluded volume (see insets in Figure 4a), Figure 4c presents results only for *R* = 0. The dependence of *P*_rk_ on *u* for 5_1_ knots (blue line) qualitatively reproduces the trends observed for 3_1_ knots: we see pronounced excess (depletion) of right-handed knots for *u* > 0 (*u* < 0), which saturates at high *u*. This behavior is consistent with 5_1_ knot being a torus knot which (for open chains) can be obtained from a 3_1_ knot by one extra winding around the knot contour. Intriguingly, however, 5_2_ knots (green line) show an opposite trend; there is a surplus of right-handed knots for *u* < 0 and a depletion for *u* > 0. This, at first glance, unexpected behavior of 5_2_ knots demonstrates that the handedness of knots may not coincide with the helical sense of the molecule.

We suggest that the coupling between helicity and topological chirality found in our simulations stems (to large extent, at least) from a generic mechanism. Namely, it is caused by conformations where some of the knot crossings are encapsulated in a “braid” formed by two interwoven helical subchains. Figure 5a illustrates three representative conformations of 3_1_, 5_1_, and 5_2_ knots with such a braid (the braided part is enlarged, in a separate frame) for *R* = 0 (for clarity, we are showing only the knotted part of an *N* = 2000 chain). The sense (direction) of winding of the subchains around each other is the same as the sense of the polymer helix (positive in the examples of Figure 5a).

**Figure 5:**
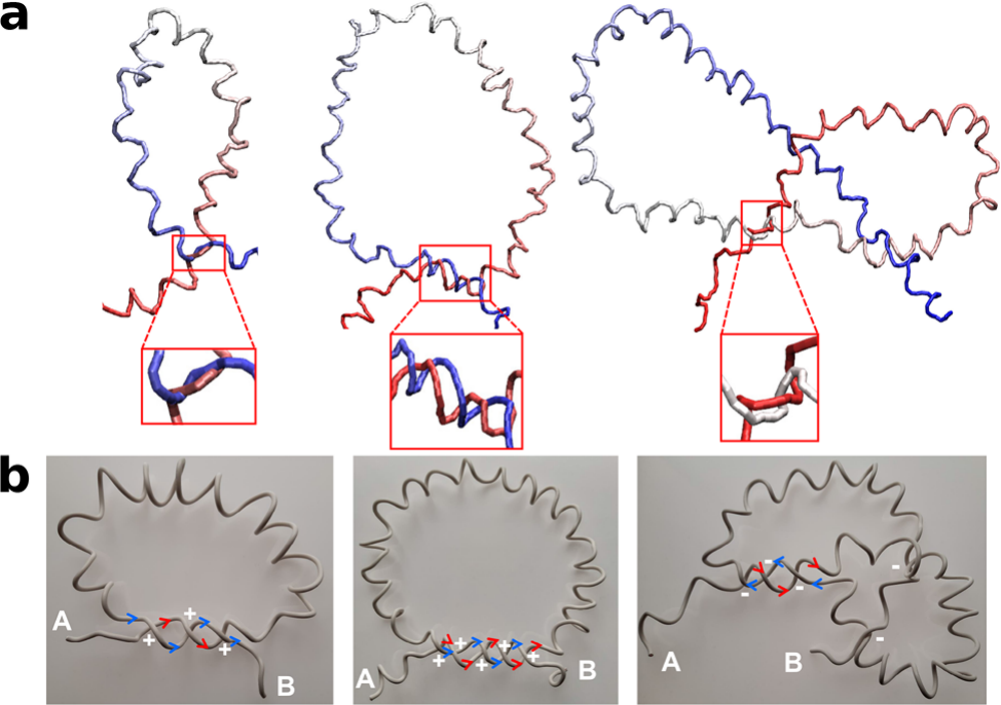
(a) Snapshots of parts of an *N* = 2000 chain forming 3_1_, 5_1_, and 5_2_ knots (from left to right) for *u* = 0.5 and *R* = 0, illustrating typical shapes of knots with braids (for these parameters). The braided part of the knots is shown enlarged, in a separate frame. (b) Illustrations of the same knots based on an idealized physical (wire) model. The chain contour is traced from end A toward end B. Red and blue arrows, respectively, mark the direction of motion along the first and the second polymer strand forming the braid. The topology of 3_1_ and 5_1_ knots is such that the two strands are traveled in the same direction, whereas for the 5_2_ knot, they are traveled in opposite directions. This difference affects the handedness of the crossings, which is indicated by the ± signs.

Initially, our explanation is motivated by visual inspection of knotted conformations. Of course, visual analysis cannot be systematic because of the significant amount of knotted conformations and their variability. However, there are also several more quantitative arguments that favor our conjecture based on indirect evidence. First of all, helices, say, with positive helical sense, interwoven into a braid with positive twist can *simultaneously* explain the preference for *positive* handedness in 3_1_ and 5_1_ and the *negative* handedness in 5_2_ knots, respectively. Figure 5b provides explanatory illustrations based on an idealized physical (wire) model of helical knotted chains. These illustrations may also indicate that for large |*u*| torus knots, 3_1_ and 5_1_ are easier to form and have a simpler braiding pattern than, for example, a 5_2_ knot, providing an interpretation for results observed in Figure 4b. A similar analysis of knots in terms of “braids” was performed^56,57^ to explain the occurrence of certain knot types in template synthesis of molecular knots.^58^ Second, we take into account that in interwoven helices segments come close to each other. Therefore, for *R* = 0, we eliminate from the sample knotted conformations where at least two segments (separated by more than *d* segments along the WLC contour) are found closer than a cutoff distance *r*_min_; a typical choice is *r*_min_ = 1.5. For knots that survive this screening (and therefore have no tightly packed braids), the deviations of *P*_rk_ from 50% are significantly reduced. Plots are available in the Supporting Information. Consistent with the effect of screening for *R* = 0, we observe a reduced deviation of *P*_rk_ from 50% in systems with excluded volume, especially *R* = 2.5 (Figure 4c). In the latter case, compact helices do not allow for molecular interdigitation sufficient for forming braids. Finally, we note that for *R* = 0.5 and 2.5 there is also a similarity between the nonmonotonous dependence of *P*_rk_ on *u* and the behavior of *C*_c_ in Figure 3b. This observation suggests that strong fluctuations in local chain conformations and helicity promote interdigitation.

Various studies^59−63^ have revealed that special packing of molecules with helical surface affects mesoscopic chiral order in multichain systems. In this respect, our observations regarding the relationship between local packing of helices and topological chirality of knots are not surprising. Still, it is rather unexpected that the fraction of conformations found in this particular knotted state is sufficiently large to cause perceptible mirror-symmetry breaking for the entire set of 3_1_, 5_1_, and 5_2_ knots. Detailed analysis of knot handedness versus the amount of monomers contained in a knot demonstrates that broken mirror symmetry is more pronounced for smaller knots. Hence, it is plausible to expect that the effects of helicity on topological chirality of the entire population of knots in longer chains (than those that have been considered here) will be reduced.

Our findings are based on a generic molecular model but can be extrapolated to actual helical polymers. We expect that an excess of knots with one sense of handedness might be observed in chiral polymers where helices have well-separated “ridges” and “valleys”, the analog of open helices formed in our model at a small excluded volume. Polyisocyanates^64,65^ might be one example, taking into account that the formation of their lyotropic cholesteric phases can be explained^62^ assuming a strongly corrugated, screw-like, helical molecular surface. Another candidate are biopolymers with polyproline helices of type PP-II. In contrast, we do not expect strong preferred handedness for knots in polymers with compact helices. Here, representative examples are biopolymers with polyproline helices of type PP-I or α-helices.^66^ The topology of single knotted polymer conformations can be analyzed by modern imaging techniques such AFM.^67^

## Supporting Information Available

Details on the applied reptation algorithm; The effect of exclusion of conformations with closely packed segments on handedness of trefoil knots; The effect of polymer helicity on the prevalent type of fivefold knots (PDF)
