# Supplementary material for: Can Polymer Helicity Affect Topological Chirality of Polymer Knots?
Source: ACS Macro Lett. 2023 Jan 27;12(2):234–40. doi: 10.1021/acsmacrolett.2c00600 (PMC9948535; doi:10.1021/acsmacrolett.2c00600)
Supplement: Supplementary file 1 — mz2c00600_si_001.pdf [file mz2c00600_si_001.pdf]

Supporting Information for: Can polymer helicity affect topological chirality of polymer knots?

Yani Zhao,[†] Jan Rothörl,[‡] Pol Besenius,[¶] Peter Virnau,[*,‡] and Kostas Ch. Daoulas[*,†]

†*Max Planck Institute for Polymer Research, Ackermannweg 10, 55128 Mainz, Germany*

‡*Department of Physics, Johannes Gutenberg University Mainz, Staudinger Weg 9, 55128 Mainz, Germany*

¶*Department of Chemistry, Johannes Gutenberg University Mainz, Duesbergweg 10-14, 55128 Mainz, Germany*

E-mail: virnau@uni-mainz.de; daoulas@mpip-mainz.mpg.de

1 Details on reptation algorithm

We use Metropolis Monte Carlo sampling based on a variant of the standard reptation algorithm.[1,2]

The basic idea behind the reptation algorithm[1,2] is to propose to the Metropolis acceptance criterion new polymer configurations, generated by "cutting" a number of segments from one chain end and rebuilding them at the opposite chain end. Since we are dealing with isolated chains, described by a simple molecular model, we can achieve reasonable acceptance rates by cutting and regrowing two segments at each reptation "move". We note that for dense systems, e.g. melts, described by all-atom models, reptation algorithms frequently "displace" only single segments[2,3]

(unless advanced biased sampling is used during chain reconstruction, e.g. configurational bias[4,5]).

In terms of the worm-like chain model (WLC) used in our simulations, the algorithm is fomulated as follows:

Step 1 Start from an initial configuration where the WLC with N segments is completely straight, i.e. a "line".

Step 2 Select with equal probability one of the two ends of the WLC and "cut" the two last segments of the WLC that are found at this end. Re-attach these two segments at the other end of the chain. The re-attachment proceeds as follows. Let the Cartesian coordinates of the other end be $x_{\rm e}$, $y_{\rm e}$, $z_{\rm e}$. Generate two random numbers $\xi_1, \xi_2 \in [0,1)$ and define two variables $\phi = 2\pi\xi_1$ and $\chi = 1-2\xi_2$. ϕ has the meaning of the azimuthal angle of the bond and χ has the meaning of the cosine of the polar angle θ of the bond (with respect to the z-axis). The length of the bond is fixed to b. Then the new coordinates of the first segment are given by $x_{\rm n1} = x_{\rm e} + b\chi$, $y_{\rm n1} = y_{\rm e} + b\sqrt{1-\chi^2}\cos(\phi)$ and $z_{\rm n1} = z_{\rm e} + b\sqrt{1-\chi^2}\sin(\phi)$. The new position of the second segment is generated in a similar way: two random numbers are generated, the variables $\tilde{\phi}$ and $\tilde{\chi}$ are defined, and the new coordinates of the second segment are given by $x_{\rm n2} = x_{\rm n1} + b\tilde{\chi}$, $y_{\rm n2} = y_{\rm n1} + b\sqrt{1-\tilde{\chi}^2}\cos(\tilde{\phi})$ and $z_{\rm n2} = z_{\rm n1} + b\sqrt{1-\tilde{\chi}^2}\sin(\tilde{\phi})$.

Step 3 The new conformation is accepted with probability $p_{\rm acc} = \min\{1, e^{\frac{-(E_{\rm new}-E_{\rm old})}{k_BT}}\}$, i.e. following the standard Metropolis acceptance criterion. Here $E_{\rm new}$ and $E_{\rm old}$ are, respectively, the total energy of the new and the old conformation. We note that for those cases where we have excluded volume, $R > 0$, the proposed conformation is rejected straightaway in case the segments in the new position overlap with other segments of the WLC.

Step 4 If the proposed conformation is rejected, both segments remain in their old position and we move again to Step 2. If the proposed conformation is accepted, the two displaced segments are assigned their new position. After standard "bookkeeping" operations, e.g. update of neighbour lists, we move again to Step 2.

Step 5 Repeat the steps 2-4 until a sufficiently large set of decorrelated conformations is generated.

We estimate decorrelation times in a standard way, from the decay of the chain end-to-end vector orientational autocorrelation function.[3] For the systems considered in our study, the polymers with $N = 2000$, $R = 2.5$, and $u = \pm 0.5$ have the longest decorrelation time which corresponds, roughly, to 420 millions of attempted reptation moves. We note that for this set of parameters, the reptation move has the lowest acceptance rate, 3.5% (comparing to systems with smaller u and/or smaller R considered in our study).

2 Closely-packed segments affect handedness of trefoil knots

Figure S1 shows the probability $P_{\rm rk}$ that a knot formed at given u is right handed ($P_{\rm rk} = 50\%$ indicates no preferred handedness) without or with a cutoff distance. The cutoff is applied to remove knots with tightly packed braids. For a given cutoff distance $r_{\rm min}$, $P_{\rm rk}$ is calculated after excluding knotted conformations with segments that are closer than $r_{\rm min}$. For chains without excluded volume, $R = 0$, the deviations of $P_{\rm rk}$ from 50% are significantly reduced when the cutoff is introduced (Figure S1a). Similar effect albeit more attenuated is observed for chains with an excluded volume of $R = 0.5$ (Figure S1b).

3 Helicity affects the prevalent type of fivefold knots

The ratio of 5_1 and 5_2 knots in Figure S2 shows how helicity impacts the formation of different knot types. 5_1 knots are favored for large values of $|u|$ while 5_2 knots are favored around $|u| = 0$.

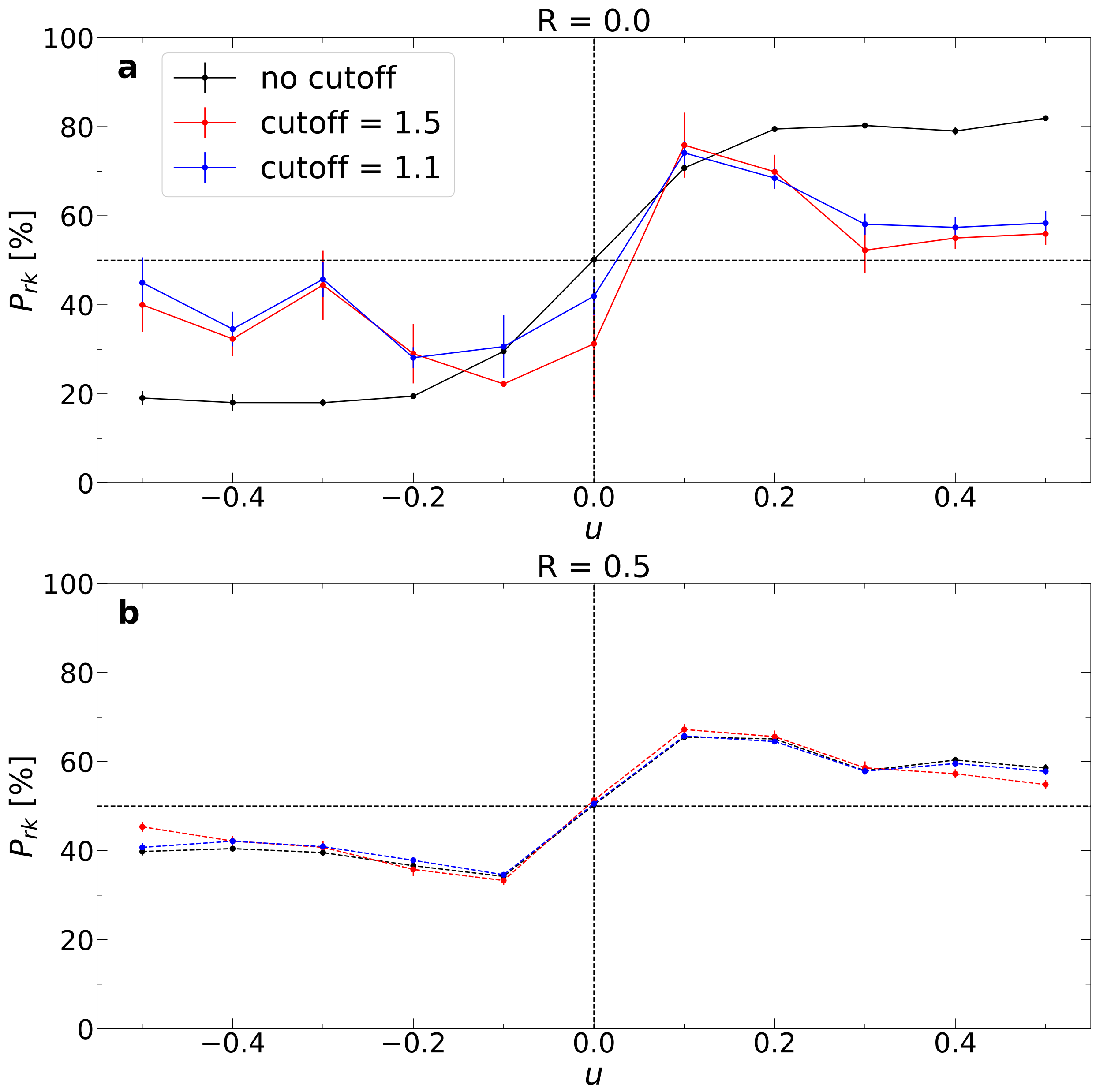

Figure S1: (a) Handedness of trefoil knots without excluded volume. The black line is the same as in Figure 4 in the main text. The red and blue lines are the results from different choices of distances between WLC segments used to exclude knots with tightly packed braids. Here, all knots containing beads closer than $r_{ij} = 1.1$ or 1.5 to each other, inside the knotted part of the chain, are ignored. This screening reduces the excess of handedness in the remaining knots indicating that knots with tight braids are indeed one mechanism leading to an excess of one kind of handedness. The error was calculated by splitting the recorded data into ten uncorrelated blocks of the same size and then calculating the standard error of the mean. The larger error for negative u compared to positive u is due to less recorded statistics in the former case. (b) Same plot for chains where segments have an excluded volume of $R = 0.5$, indicating an effect similar to (a).

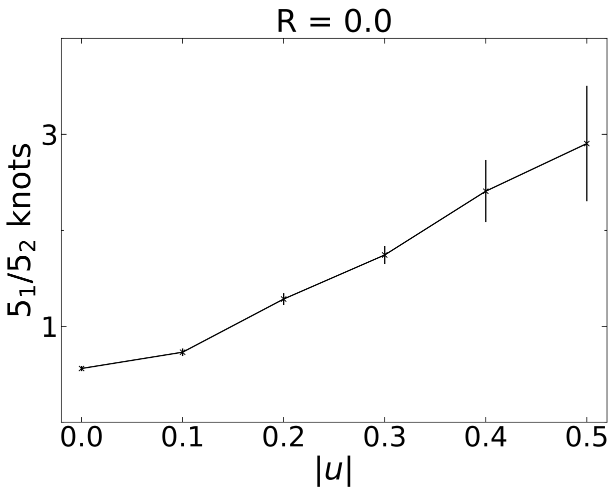

Figure S2: Ratio of the occurrence of 5_1 and 5_2 knots in chains with no excluded volume, $N = 2000$ and different $|u|$. One can see that increasing $|u|$ leads to an excess of 5_1 knots compared to 5_2 knots. The error was calculated by splitting the recorded data into ten uncorrelated blocks of the same size and then calculating the standard error of the mean.

References

(1) Wall, F.; Mandel, F. Macromolecular Dimensions Obtained by an Efficient Monte Carlo Method without Sample Attrition. *J. Chem. Phys.* **1975**, *63*, 4592–4595.

(2) Vacattelo, M.; Avitabile, G.; Corradini, P.; Tuzi, A. A Computer Model of Molecular Arrangement in a n-paraffinic liquid. *J. Chem. Phys.* **1980**, *73*, 548–552.

(3) Mavrantzas, V. G.; Boone, T. D.; Zervopoulou, E.; Theodorou, D. N. End-Bridging Monte Carlo: A Fast Algorithm for Atomistic Simulation of Condensed Phases of Long Polymer Chains. *Macromolecules* **1999**, *32*, 5072–5096.

(4) de Pablo, J. J.; Laso, M.; Suter, W. U. Estimation of the Chemical Potential of Chain Molecules by Simulation. *J. Chem. Phys.* **1992**, *96*, 6157–6162.

(5) Siepmann, J. I.; Frenkel, D. Conflgurational Bias Monte Carlo: A New Sampling Scheme for Flexible Chains. *Mol. Phys.* **1992**, *75*, 59–70.
